# The CXCR3(+)CD56Bright Phenotype Characterizes a Distinct NK Cell Subset with Anti-Fibrotic Potential That Shows Dys-Regulated Activity in Hepatitis C

**DOI:** 10.1371/journal.pone.0038846

**Published:** 2012-07-05

**Authors:** Marianne Eisenhardt, Andreas Glässner, Benjamin Krämer, Christian Körner, Bernhard Sibbing, Pavlos Kokordelis, Hans Dieter Nischalke, Tilman Sauerbruch, Ulrich Spengler, Jacob Nattermann

**Affiliations:** Department of Internal Medicine I, University of Bonn, Bonn, Germany; Karolinska Institutet, Sweden

## Abstract

**Background:**

In mouse models, natural killer (NK) cells have been shown to exert anti-fibrotic activity via killing of activated hepatic stellate cells (HSC). Chemokines and chemokine receptors critically modulate hepatic recruitment of NK cells. In hepatitis C, the chemokine receptor CXCR3 and its ligands have been shown to be associated with stage of fibrosis suggesting a role of these chemokines in HCV associated liver damage by yet incompletely understood mechanisms. Here, we analyzed phenotype and function of CXCR3 expressing NK cells in chronic hepatitis C.

**Methods:**

Circulating NK cells from HCV-infected patients (n = 57) and healthy controls (n = 27) were analyzed with respect to CXCR3 and co-expression of different maturation markers. Degranulation and interferon-γ secretion of CXCR3(+) and CXCR3(−) NK cell subsets were studied after co-incubation with primary human hepatic stellate cells (HSC). In addition, intra-hepatic frequency of CXCR3(+) NK cells was correlated with stage of liver fibrosis (n = 15).

**Results:**

We show that distinct NK cell subsets can be distinguished based on CXCR3 surface expression. In healthy controls CXCR3(+)CD56Bright NK cells displayed strongest activity against HSC. Chronic hepatitis C was associated with a significantly increased frequency of CXCR3(+)CD56Bright NK cells which showed impaired degranulation and impaired IFN-γ secretion in response to HSC. Of note, we observed intra-hepatic accumulation of this NK cell subset in advanced stages of liver fibrosis.

**Conclusion:**

We show that distinct NK cell subsets can be distinguished based on CXCR3 surface expression. Intra-hepatic accumulation of the functionally impaired CXCR3(+)CD56Bright NK cell subset might be involved in HCV-induced liver fibrosis.

## Introduction

Hepatitis C Virus (HCV) is a major cause for chronic inflammatory liver disease with variable progression towards liver fibrosis/cirrhosis. Hepatic infiltration of immunocompetent cells, including lymphocytes, is a hallmark of HCV infection, and there is accumulating evidence that this inflammatory infiltrate pivotally modulates immunopathogenesis of hepatitis C.

Recruitment of lymphocytes to the liver is importantly regulated via chemokines and chemokine receptors. Chemokines are small molecules involved in the regulation of chemotaxis and tissue extravasation of lymphocytes as well as in modulation of leukocyte function. Leukocytes sense chemokine concentration gradients via their respective chemokine receptors and move towards increasing concentration gradients.

In hepatitis C the CXC-chemokine receptor CXCR3 and its ligands (CXCL9, CXCL10, CXCL11) have gained specific attention because various studies demonstrated elevated levels of CXCR3 ligand CXCL10 to be predictive of the failure to response to HCV therapy [1]–[4] and to be associated with stage of fibrosis [4]–[10].

For years it was an unsolved paradox of why a pro-inflammatory chemokine, responsible for the hepatic recruitment of activated lymphocytes, is a marker for treatment failure and advanced liver fibrosis. However, recently Casrouge and co-workers provided an interesting explanation of this phenomenon by showing that CXCL10 in the plasma of patients with chronic hepatitis C mainly exists in a truncated antagonist form [11]. This CXCL10 variant can bind to CXCR3 without signaling and competitively inhibit binding of the agonist form of CXCL10, thereby interfering with proper recruitment of CXCR3-expressing lymphocytes.

However, analyzing fibrotic livers from HCV-infected patients Zeremski et al. nicely showed that most intra-hepatic lymphocytes express CXCR3 [8]. Therefore, we speculated that the mechanism(s) how CXCR3/CXCR3 ligands modulate hepatic fibrogenesis may imply biological functions beyond immune cell recruitment. Regarding hepatic trafficking of leukocytes most studies focused on the potential role of CXCR3-expressing T lymphocytes [12], [13]. However, CXCR3 is expressed on various immunocompetent cells including natural killer (NK) cells, which represent an important component of the intra-hepatic lymphocyte pool. In contrast to the peripheral blood which contains about 5–10% NK cells, intra-hepatic lymphocytes comprise about 30% NK cells, and the percentage of intra-hepatic NK cells may even increase in inflammatory liver diseases [11].

In mouse models NK cells have been shown to exert anti-fibrotic capacity [14], [15] and dys-function of NK cells was associated with a more rapid progression of liver fibrosis [16]. Accordingly, in vitro activity of NK cells has been demonstrated to correlate with the degree of HCV-associated liver fibrosis [17], [18].

However, it remained unclear whether distinct NK cell subsets differ regarding their anti-fibrotic activity.

Here, we compared phenotype and functional activity against primary human hepatic stellate cells of CXCR3(+) NK cell subsets in healthy individuals and chronic hepatitis C.

## Results

### CXCR3 Expression Dissects CD56Dim and CD56Bright NK Cells in Specific Subsets

**Figure 1:**
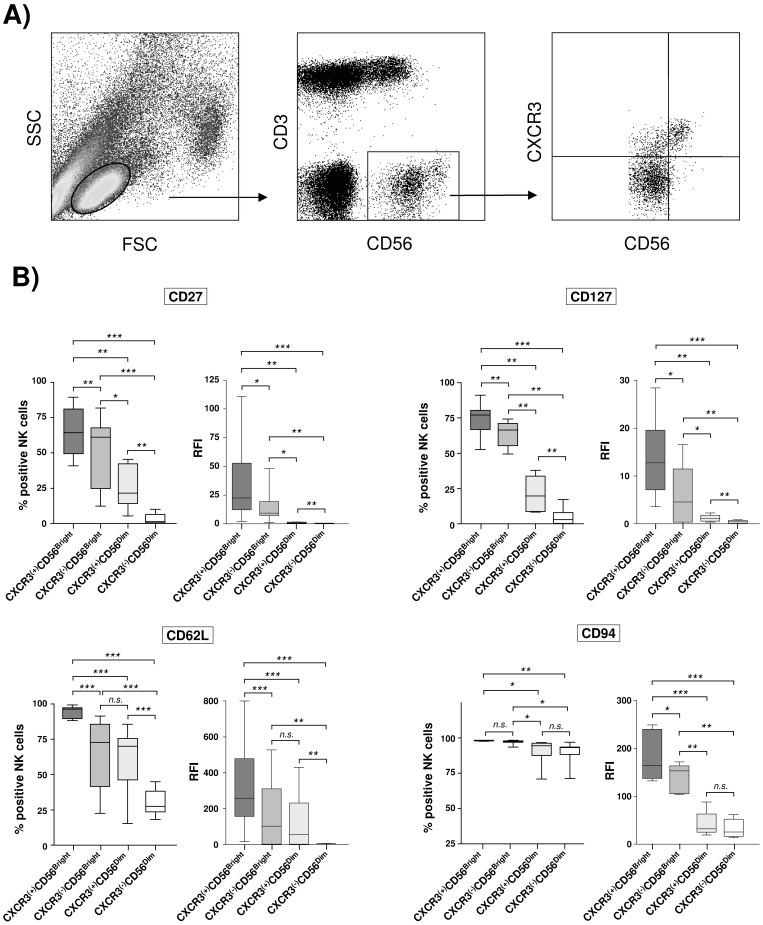
CXCR3 expression dissects phenotypically distinct NK cell subsets. Figure 1A: PBMCs in whole blood specimen were stained with anti-CD3, anti-CD56, and anti-CXCR3. CD3^(−)^ CD56^(+)^ NK cells were then gated for quantification of CXCR3 surface expression. Figure 1B: PBMCs from at least eight healthy donors were stained with anti-CD3, anti-CD56, and anti-CXCR3–conjugated mAb, as well as a mAb directed against the indicated maturation markers. CXCR3(+) and CXCR3(−) NK cell populations were then assessed for surface expression of the respective maturation marker. Results are given as box and whisker plots, with medians and 10th, 25th, 75th, and 90th percentiles. * indicates p<0.05; **indicates p<0.01; *** indicates p<0.001.

First, we studied expression of CXCR3 on circulating NK cells obtained from healthy donors. Flowcytometric analysis revealed that CD56Dim and CD56Bright NK cells can clearly be separated into CXCR3(+) and CXCR3(−) NK cell subsets (Figure 1A) with frequency of CXCR3(+) NK cells being higher in the CD56Bright sub-population.

To further characterize CXCR3(+) and CXCR3(−) NK cell sub-populations we next studied the co-expression of known NK cell maturation/differentiation markers in peripheral NK cells obtained from HCV-negative individuals. As is shown in figure 1B we found CD27, CD62L, CD127, and CD94 to be highly expressed in circulating CXCR3(+)CD56Bright NK cells, indicating an immature phenotype of this NK cell subset. Interestingly, expression of these markers progressively decreased in CXCR3(−)CD56Bright, CXCR3(+)CD56Dim, and CXCR3(−)CD56Dim NK cells, with CXCR3(−)CD56Dim NK cells displaying lowest surface expression. These findings were confirmed when NK cells from HCV infected patients were analyzed (data not shown). Taken together, these data indicate that CXCR3 expression dissects both CD56Dim and CD56Bright NK cells in phenotypically different subsets.

### CXCR3 Expression Correlates with NK Cell Activity Against Human Hepatic Stellate Cells

**Figure 2:**
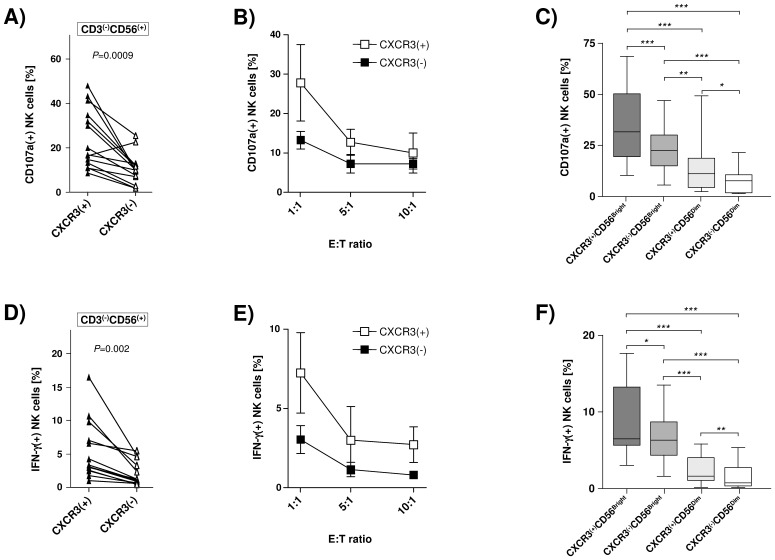
CXCR3(+)CD56Bright NK cells have strong anti-fibrotic potential. Figure 2A: Purified peripheral NK cells from healthy individuals (n = 14) were co-incubated with primary human hepatic stellate cells (E:T ratio 1∶1) and then analyzed with respect to degranulation (CD107a) and expression of CXCR3 by flowcytometry. Figure 2B depicts NK cell degranulation following co-incubation with HSC at different effector : target (E:T) ratios as indicated (n = 5). Figure 2C shows CD107a expression of the four studied NK cell sub-populations obtained from healthy donors (n = 14) following co-incubation with primary hepatic stellate cells (E:T ratio 1∶1). Figure 2D illustrates IFN-γ production of purified CXCR3(+) and CXCR3(−) NK cells obtained from HCV(-) individuals (n = 14) after co-culturing with primary HSC (E:T ratio 1∶1). Figure 2E shows IFN-γ production of purified NK cells following co-incubation with hepatic stellate cells at different E:T ratios (n = 4). Figure 2F displays IFN-γ production of the four studied NK cell sub-populations isolated from healthy donors (n = 14) following co-incubation with primary hepatic stellate cells (E:T ratio 1∶1).

CXCR3/CXCR3 ligands have been suggested to play a role in HCV-associated hepatic fibrogenesis. Therefore, we next analyzed the functional activity of circulating CXCR3(+) and CXCR3(−) NK cells using primary human hepatic stellate cells (HSC) as targets. We found that peripheral CXCR3-positive NK cells from healthy individuals displayed significantly higher degranulation as compared to their CXCR3-negative counterparts following co-incubation with HSC (Figure 2A). To verify that this differential functional activity was correlated with CXCR3 expression we then studied CD56BrightCXCR3(+), CD56BrightCXCR3(−), CD56DimCXCR3(+), and CD56DimCXCR3(−) NK cells separately (Figure 2C). Of note, we observed a sequential decrease in degranulation in these four subsets with highest activity in CD56BrightCXCR3(+) NK cells (CD56BrightCXCR3(+) > CD56BrightCXCR3(−) > CD56DimCXCR3(+) > CD56DimCXCR3(−).

Beyond direct killing of HSC NK cells have been suggested to mediate anti-fibrotic effects via release of IFN-γ, thereby inducing HSC cell cycle arrest and apoptosis [19]. Therefore, we next studied production of IFN-γ following co-incubation with activated NK cells. As is shown in figure 2D we found CXCR3-expressing NK cells to produce significantly more IFN-γ than NK cells negative for CXCR3. Analyzing HSC-induced IFN-γ production in circulating CD56BrightCXCR3(+), CD56BrightCXCR3(−), CD56DimCXCR3(+), and CD56DimCXCR3(−) NK cells separately confirmed the sequential decrease in functional activity in these four subsets (Figure 2F).

### Expression of NKG2D on CXCR3(+) and CXCR3(−) NK Cell Subsets

**Figure 3:**
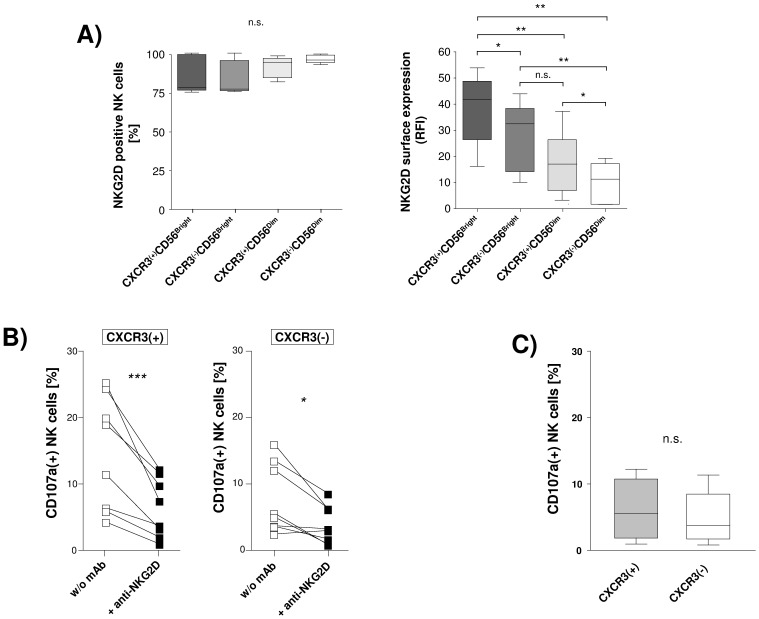
Role of NKG2D in anti-fibrotic activity of CXCR3-expressing NK cells. Figure 3A: Circulating NK cells obtained from HCV(-) individuals (n = 5) were analyzed for co-expression of NKG2D and CXCR3. The figure compares frequency of NKG2D-positive cells (left graph) as well as density of NKG2D surface expression (relative fluorescence intensity, RFI) (right graph) between the four studied NK cell subsets. Figure 3B shows the effect of NKG2D blockade on HSC-induced degranulation of CXCR3(+) and CXCR3(−) CD56Bright NK cell subsets obtained from healthy donors (n = 8). Figure 3C compares HSC-induced degranulation of CXCR3(+) and CXCR3(−) CD56Bright NK cell subsets (n = 8) following pre-incubation with anti-NKG2D. Results are given as box and whisker plots, with medians and 10th, 25th, 75th, and 90th percentiles. * indicates p<0.05; **indicates p<0.01; *** indicates p<0.001.

The activating NK cell receptor NKG2D has been shown to be critically involved in killing of hepatic stellate cells by NK cells. Thus, we next studied expression of this NK cell receptor in circulating CXCR3-expressing and CXCR3-negative NK cell subsets. Frequency of NKG2D(+) NK cells did not differ between the four studied NK cell subsets. However, we found CXCR3(+) NK cells to display a significantly higher NKG2D surface expression as compared to their CXCR3(−) counterpart (Figure 3A).

Thus, we next performed blocking experiments to confirm a functional role of NKG2D in NK cell-mediated killing of HSC. Indeed, we found that co-incubation with a NKG2D-specific blocking antibody significantly reduced NK cell degranulation following co-incubation with HSC in both CXCR3(+) and CXCR3(−)CD56Bright NK cells (Figure 3B). A role for NKG2D was furthermore supported by the fact that cytotoxic activity of CXCR3(+) and CXCR3(−) CD56Bright NK cells against HSC did not differ significantly when NKG2D was blocked with a specific antibody (Figure 3C).

### CXCR3(+)CD56Bright NK Cells from HCV-positive Patients Show Impaired Activity Against Hepatic Stellate Cells and Accumulate in the Fibrotic Liver

Finally, we studied whether chronic HCV infection might affect phenotype and function of CXCR3-expressing NK cells.

**Figure 4:**
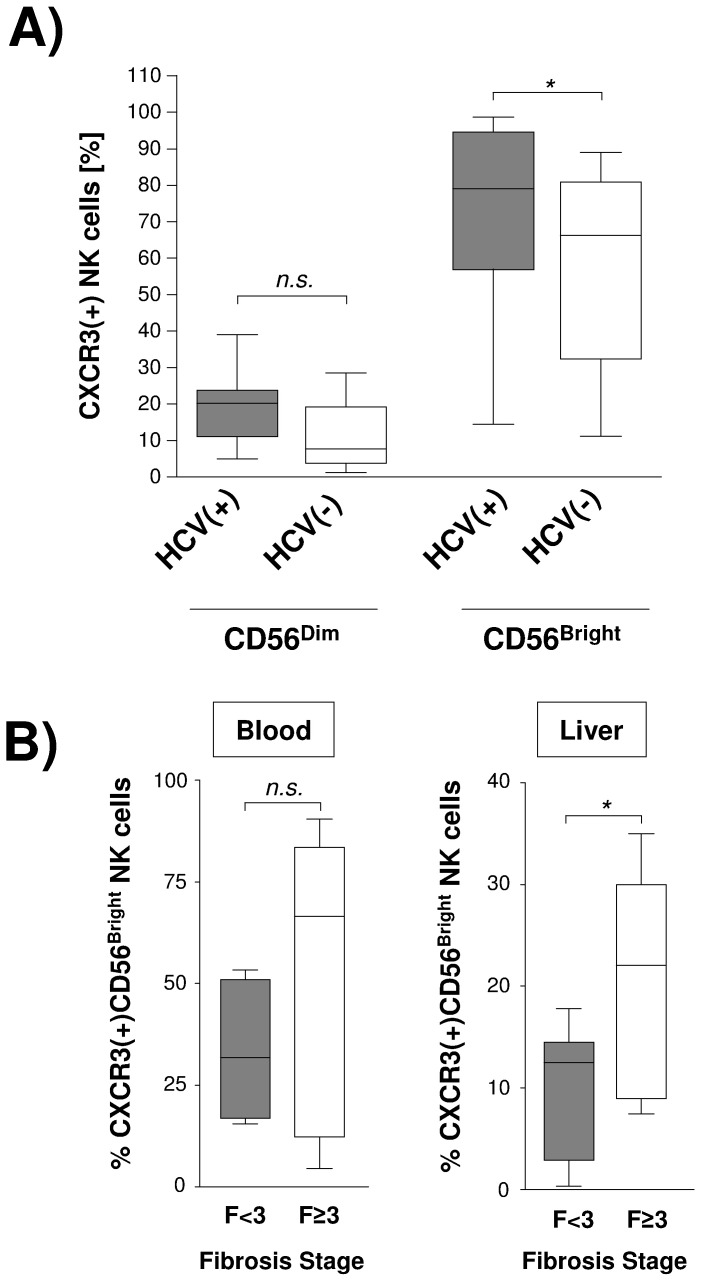
Frequency of CXCR3 expressing NK cells in chronic hepatitis C. Figure 4A compares frequency of peripheral CXCR3(+) NK cells in HCV infected (n = 14) and healthy individuals (n = 16). Figure 4B: Peripheral and liver-infiltrating NK cells from HCV-infected individuals were analyzed for expression of CXCR3 by flowcytometry Then frequency of circulating (left graph) and intra-hepatic (right graph) CXCR3(+)CD56Bright NK cells was compared between patients with progressive (F≥3; n = 6) and less advanced (F<3; n = 9) liver fibrosis. Results are given as box and whisker plots, with medians and 10th, 25th, 75th, and 90th percentiles. * indicates p<0.05.

Analyzing circulating NK cells obtained from HCV infected patients confirmed the dichotomous pattern of CXCR3 expression. However, chronic hepatitis C was associated with a significantly increased percentage of peripheral CXCR3(+)CD56Bright NK cells as compared to HCV(−) controls (Figure 4A).

Dys-regulated hepatic recruitment of CXCR3-expressing immunocompetent cells has been suggested as a potential mechanism involved in the observed association between levels of CXCR3 ligands and stages of HCV-induced liver fibrosis. Thus, we next studied intra-hepatic frequency of CXCR3(+)CD56Bright NK cells in patients showing different degrees of liver fibrosis. Given the strong anti-fibrotic potential of peripheral CXCR3(+)CD56Bright NK cells observed in healthy individuals we speculated that advanced stages of HCV-associated liver fibrosis may be associated with a decreased intra-hepatic frequency of this NK cell subset. Surprisingly, the opposite was true as we found a significantly higher frequency of CXCR3(+)CD56Bright NK cells in livers of patients with F3/4 fibrosis as compared to livers with less advanced fibrosis (Figure 3B).

**Figure 5:**
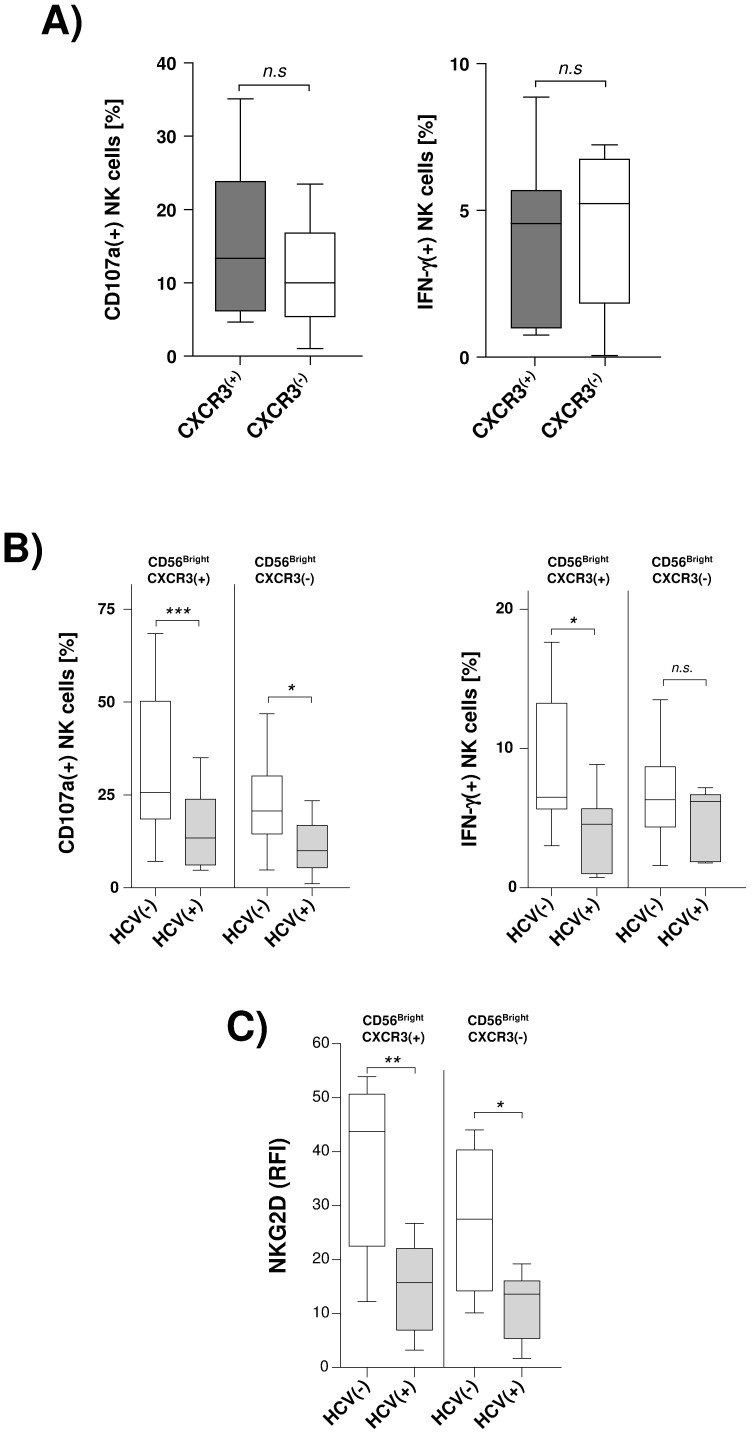
CXCR3(+)CD56Bright NK cells show impaired functional activity against HSC in chronic hepatitis C. Figure 5A: Circulating CXCR3(+) and CXCR3(−) CD56Bright NK cells from HCV(+) patients (n = 11) were co-incubated with primary human hepatic stellate cells and then assessed for degranulation (CD107a) as well as IFN-γ production by FACS analysis. Figure 5B compares HSC-induced degranulation and IFN-γ secretion between NK cell subsets obtained from healthy (n = 14) and HCV(+) (n = 11) donors. Figure 5C illustrates NKG2D surface expression on circulating CXCR3(+) and CXCR3(−) CD56Bright NK cells in hepatitis C (n = 7) in comparison to healthy controls (n = 4). Results are given as box and whisker plots, with medians and 10th, 25th, 75th, and 90th percentiles. * indicates p<0.05.

To better understand this finding we next analyzed functional activity of NK cells against HSC in chronic HCV infection. Unlike natural killer cells obtained from healthy individuals we could not find any significant differences regarding HSC-induced degranulation and IFN-γ secretion between circulating CXCR3(+)CD56Bright and CXCR3(−)CD56Bright NK cell subsets in HCV-infected patients (Figure 5A). More importantly, peripheral CXCR3(+)CD56Bright NK cells obtained from HCV(+) patients exhibited significantly lower HSC-induced degranulation (p = 0.009) and IFN-γ production (p = 0.02) than CXCR3(+)CD56Bright NK cells from healthy controls (Figure 5B). Impaired cytotoxic activity was also found in the other NK cell subset. However, significantly decreased production of IFN-γ was a specific finding in the CXCR3(+)CD56Bright subset, suggesting that CXCR3(+)CD56Bright NK cells might represent a NK cell subset with dys-regulated anti-fibrotic potential in chronic hepatitis C. As a potential mechanism underlying this impaired functional activity we found chronic hepatitis C to be associated with a significantly decreased surface expression of NKG2D (Figure 5C).

## Discussion

Infection with the hepatitis C virus often results in chronic liver disease and subsequent development of liver fibrosis/cirrhosis.

The exact mechanisms leading to liver injury are only partly understood. However, there is clear evidence indicating that activation of the immune response is a critical factor for the pathogenetic processes leading to progressive tissue injury and ultimately cirrhosis. Accordingly, liver cell damage has been shown to be associated with the presence of an intra-hepatic inflammatory infiltrate. On the other hand, there is increasing data suggesting that natural killer cells, a major component of the intra-hepatic lymphocyte pool, may mediate anti-fibrotic effects. In mouse models NK cells have been shown to exert anti-fibrotic activity by killing activated stellate cells. Moreover, impaired activity of murine NK cells has been associated with progressive liver fibrosis [14], [15].

Hepatic recruitment of lymphocytes such as NK cells is regulated by chemokines and their respective receptors. Lymphocytes sense chemokine concentration gradients and move toward increasing concentrations. Thus, both differential chemokine secretion in the inflamed liver and selective expression of different chemokine receptors on distinct lymphocyte subsets regulate hepatic recruitment of lymphocytes [2]. Of note, recent data indicate that the chemokines receptor CXCR3 and its ligands, especially CXCL10 (IP-10) may play an important role in the regulation of HCV-associated liver fibrosis [5]–[8] by yet incompletely understood mechanisms. Given the observed anti-fibrotic potential of NK cells we speculated that CXCR3-mediated recruitment of functionally distinct NK cell subpopulation might play a role.

To clarify this issue, we first analyzed the *ex vivo* phenotypic characteristics of circulating CXCR3(+) and CXCR3(−) NK cells. CXCR3-expressing NK cells were found in both the CD56Dim and the CD56Bright subset although frequency of CXCR3(+) NK cells was higher among CD56Bright cells. More importantly, comparing CD56BrightCXCR3(+), CD56BrightCXCR3(−), CD56DimCXCR3(+), and CD56DimCXCR3(−) NK cells separately we observed a progressive decrease in surface expression of the maturation markers CD27, CD62L, and CD127. In addition, the CXCR3(+) subset displayed significantly higher co-expression of the NK cell receptors NKG2A, NKG2C, and NKp44. Thus, our data suggest that expression of CXCR3 defines distinct NK cell subsets.

This concept was confirmed at the functional level, because in healthy controls CXCR3(+) and CXCR3(−) NK cell subsets differed significantly with respect to cytolytic activity and IFN-γ production after exposure to human hepatic stellate cells, with CD56BrightCXCR3(+) NK cells displaying the strongest activity against HSC. Activated HSC are considered to critically contribute to the establishment of hepatic fibrosis via excessive production of collagen. Thus, effective killing of HSC by CD56BrightCXCR3(+) NK cells suggests that this specific lymphocyte subset may play an important role in the regulation of HCV-associated liver fibrosis. However, in hepatitis C high levels of CXCR3 ligands have been associated with progressive liver cell damage and fibrosis [5]–[8]. Recently, these counterintuitive data could – at least in part – be explained by the identification of a chemokine antagonism in HCV infection which prevents CXCR3-mediated recruitment of immunocompetent cells into the liver thereby resulting in extra-hepatic accumulation of CXCR3-expressing cells [11]. Accordingly, we found chronic hepatitis C to be associated with a significantly increased frequency of circulating CXCR3-expressing CD56Bright NK cells. However, we also observed a significantly increased frequency of CXCR3(+)CD56Bright NK cells in livers with advanced stages of fibrosis, suggesting that mechanism(s) other than dys-regulated hepatic migration may also play a role.

Indeed, our data indicate that hepatitis C may be associated with impaired activity of peripheral CXCR3(+) NK cells because we found that in HCV infected patients_specifically the CD56BrightCXCR3(+) subset displayed decreased degranulation and IFN-γ secretion in response to human HSC. Unfortunately, number of intra-hepatic NK cells in our study was insufficient to study activity of liver NK cells against HSC. Thus, it remains to be clarified whether intra-hepatic CXCR3-expressing NK cells also show dys-regulated activity against activated HSC or even exert pro-fibrotic effects.

The exact mechanisms responsible for this impaired functional activity of circulating CD56BrightCXCR3(+) NK cells in HCV infection remain incompletely understood but may involve altered surface expression of the NK cell receptor NKG2D. Mouse models indicate that NK cell killing of activated hepatic stellate cells is mediated via activating NK cell receptor NKG2D [14]–[16], [20]–[24]. Accordingly, we found that blocking of NKG2D significantly reduced NK cell degranulation and IFN-γ secretion following co-incubation with HSC. Thus, our finding of decreased NKG2D surface expression in hepatitis C would be an intriguing explanation for our observation of impaired NK cell activity against HSC. However, the role of NKG2D in HCV infection is discussed controversially as some authors reported down-regulated expression of this NK cell receptor [25] whereas other studies found increased surface expression in hepatitis C [26], [27]. In line with data presented by Sène and colleagues we found that differences in HCV(+) patients and healthy controls only affected expression levels of NKG2D, but not the frequency of NKG2D-positive cells [25].

However, reduced expression of NKG2D was not specific for the CXCR3(+)CD56Bright subset, indicating that other mechanisms may also play a role.

Taken together, we show that distinct NK cell subsets can be distinguished based on CXCR3 surface expression. Intra-hepatic accumulation of functionally impaired CD56BrightCXCR3(+) might be involved in the progression of HCV-induced liver fibrosis.

## Methods

### Patients

A total of 57 patients of Western European descent, all from the Bonn area in Germany, with chronic hepatitis C virus (HCV) infection were enrolled into this study (Table 1). None of these patients had histological evidence of liver cirrhosis and all were treatment naïve. As control we studied 27 healthy donors. Written informed consent was obtained from all patients. The study had been approved by the local ethics committee of the University of Bonn.

**Table 1:** Patient Characteristics.

	HCV RNA(+)	healthy controls
Number	57	27
Female sex	16 (28.1%)	13 (48.1%)
Age (years)	46.95 (17–72)	29.1 (22–49)
**Clinical data**		
ALT U/L	96.2 (13–384)	n.a. c)
γ-GT	142.9 (21–957)	n.a. c)
**HCV-Status**		-
HCV Load (×10^6^ copies/mL) b)	5.2 (n.a. –23.3)	-
HCV-Genotypes:		
Genotype 1 ^1a)^	29 (50.9%)	-
Genotype 2 ^1a)^	3 (5.3%)	-
Genotype 3 ^1a)^	8 (14.0%)	-
Genotype 4 ^1a)^	4 (7.0%)	-
Undetermined Genotype ^1a)^	13 (22.8%)	-

a) number of cases (number/total in %).

b) mean (range).

c) n.a. – not analyzed.

#### Flowcytometric Analysis

For FACS analysis the following antibodies were used: anti-CD3, anti-CD27, anti-CD56, anti-CD62L, anti-CD107a, anti-CD127 (BD Biosciences, Heidelberg, Germany); anti-CXCR3, anti-NKG2A, anti-NKG2C, anti-NKG2D, anti-NKp30, anti-NKp44, anti-NKp46, and anti-INF-γ (R&D Systems; Wiesbaden-Nordenstadt, Germany). After staining, cells were washed and analyzed on a FACSCalibur flowcytometer using the Flowjo 7.2.2 software package (Treestar, Ashland, USA).

### Cell Separation

NK cells were immunomagnetically separated from total PBMC by depletion of non-NK cells using EasySep Human NK Cell Enrichment Kit (StemCell Technologies, Grenoble, France). NK cells were cultured overnight for further experiments in RPMI1640 (PAA, Cölbe, Germany) supplemented with 25IU IL-2 (R&D Systems).

Purity of NK cells was >95% as determined by flowcytometric analysis.

### Primary Human Hepatic Stellate Cells

Isolated primary activated human hepatic stellate cells (HSC; ScienCell, San Diego, CA, USA) ^(15–17)^ were cultured for 2–4 passages in defined Stellate Cell Medium (SteCM, ScienCell) supplemented with 2% fetal bovine serum, 5 ml stellate cell growth supplement, 10 U/ml penicillin and 10 µg/ml streptomycin (all ingredients obtained from ScienCell) at 37°C with 5% CO_2_ and cryopreserved until further use.

Two days before HSC were used in an experiment the cells were thawed and cultured in SteCM medium. Then cells were harvested, washed, checked for viability using trypan blue, and then used in the respective experiments. Activated status of HSC was verified by immunofluorescence staining of α-smooth muscle actin.

### IFN-γ Production

Isolated NK cells were co-cultured with HSC at 1∶1 effector:target (E:T) ratio in 48-well round-bottom plates at 37°C for 5 h. Brefeldin A (10 µg/ml, Sigma-Aldrich) was added after 1hour of co-culture. Next, cells were harvested and washed in PBS. Finally, cells were stained with anti-CXCR3-FITC, anti–CD56 APC, and anti–CD3 PerCP, fixed and permeabilized using Cytofix/Cytoperm (BD Biosciences), followed by intracellular staining with an anti–IFN-γ PE mAb and FACS analysis.

### CD107a Degranulation Assay

Cytotoxic activity of NK cells was assessed by a CD107a degranulation assay as described before [18]. In brief, purified NK cells were co-incubated with HSC at 1∶1 effector:target (E:T) ratio in the presence of CD107a mAb. After 1 h GolgiStop (BD Biosciences) was added and cells were cultured for an additional 3 h. Then, cells were stained, washed, and re-suspended in CellFix (BD Biosciences) followed by flowcytometric analysis.

### Flow Cytometric Analysis of Cells in the Liver Specimens

Liver biopsy specimens for flowcytometric analysis of intra-hepatic cells were obtained from liver biopsies (n = 15) using 1.4 mm diameter disposable biopsy needles. Grading and staging of liver biopsies were performed according to the METAVIR score as part of the routine diagnostic work-up (F0: n = 5, F1: n = 1, F2: n = 3, F3: n = 4, F4: n = 2).

Fresh liver samples were washed twice in fresh medium and shaken gently to avoid blood contamination. Liver specimens were disrupted mechanically into small fragments in RPMI 1640 medium with 10% FCS using a forceps and scalpel. Then the fragments were homogenized on a cell strainer (BD Labware). The resulting cell suspension was washed and resuspended in RPMI 1640 medium. Intra-hepatic cells were then directly analyzed by flowcytometry.

### Statistical Analysis

Statistical analyses were performed using GraphPad Prism Version 5.0a (GraphPad Software Inc, San Diego, CA) and the SPSS 17.0 (SPSS, Chicago, IL) statistical package. Mann–Whitney *U* tests were used to compare NK cell phenotype and cytolytic response between two independent groups. Wilcoxon tests were performed for individual comparison of the paired groups.

A 2-sided *P* value <0.05 was considered significant.
